# Solitary Fibrous Tumor Generated From Uterine Corpus With Doege–Potter Syndrome in Primary Disease: A Rare Case of Recurrent Disease Without Hypoglycemia

**DOI:** 10.1155/crog/1416178

**Published:** 2026-07-13

**Authors:** Lin-jing Yuan, Ming Ni, Shi-cong Yang, Dan Liao, Jun-xiu Liu

**Affiliations:** ^1^ Department of Obstetrics and Gynecology, The First Affiliated Hospital of Sun Yat-sen University, Guangzhou, Guangdong, China, sysu.edu.cn; ^2^ Department of Obstetrics and Gynecology, Hanyang Hospital Affiliated to Wuhan University of Science and Technology, Wuhan, Hubei, China; ^3^ Department of Pathology, The First Affiliated Hospital of Sun Yat-sen University, Guangzhou, Guangdong, China, sysu.edu.cn; ^4^ Department of Anesthesiology, The First Affiliated Hospital of Sun Yat-sen University, Guangzhou, Guangdong, China, sysu.edu.cn

## Abstract

**Introduction:**

Solitary fibrous tumors (SFTs) generated from uterine corpus are extremely rare and hard to distinguish from myomas, but their prognoses are wildly different. Doege–Potter syndrome (DPS) is a form of nonislet cell tumor hypoglycemia (NITCH) caused by a SFT. Vigilance is required when a pelvic mass is found concurrent with NITCH.

**Case Presentation:**

A 65‐year‐old female with huge pelvic masses generated from uterine corpus presented with mild symptoms of transient slow response, accompanied by memory loss once, and was found to have hypoglycemia. The pituitary glucocorticoid axis, pituitary sex hormone axis, and pituitary growth hormone axis functioned normally; insulin release was normal, but relatively low insulin levels indicated suppression of secretion. Abdominal total hysterectomy and bilateral salpingo‐oophorectomy were performed, and pathology confirmed a benign SFT with DPS. Blood glucose levels after surgery are significantly elevated and more stable. This case fell into the intermediate‐risk group, but the disease recurred. Cytoreduction surgery was performed 13 months after initial treatment without DPS.

**Discussion:**

Uterine corpus–generated SFTs are hard to distinguish preoperatively. However, complete tumor resection is a key prognostic factor because SFTs have a risk of long‐term recurrence. DPS can be a differential diagnostic point, but there is no significant relation between tumor burden and recurrence.

## 1. Introduction

Solitary fibrous tumor (SFT) is a rare mesenchymal neoplasm that can originate from almost any anatomic site, with a marked tendency to recurrence [1]. The incidence rate of SFT is 2.8 per 100,000, and approximately 30% of cases originate in the pleura. Only 1% of SFTs develop in the female reproductive system [2]. The differential diagnosis of SFT of the uterine corpus is difficult due to its clinical symptoms and signs being similar to those of leiomyoma, and correct identification is a prerequisite for appropriate therapeutic strategies.

Nonislet cell tumor–induced hypoglycemia (NITCH) is a paraneoplastic syndrome. When attributed to SFTs, we call it Doege–Potter syndrome (DPS). Approximately 5%–10.4% of SFTs develop DPS, whether benign or malignant, which can be a differential diagnostic point, but there is no relationship with recurrence [3].

This report describes a rare case of uterine SFT with DPS that recurred without hypoglycemia, with the aim of increasing awareness of uterus origin SFTs and improving the patient′s prognosis.

## 2. Case Presentation

This patient provided full informed consent for this case report.

A 65‐year‐old female with no prior health issues presents with a 20‐day history of abdominal distension. During this period, she experienced transient slow response and memory loss once. Imaging shows multiple large, solid pelvic masses indicating uterine fibroids. PET‐CT with 18F‐FDG showed a mild increase in the metabolism of the solid part of the tumor; thus, a malignant mesenchymal tumor of the uterus needed to be distinguished (Figure 1). Left lateral ventricular mass approximately 3 cm in diameter considering subventricular tumor of the anterior portion of the left lateral ventricle (Figure 2A). Capillary glucose levels of 1.8–4.3 mmol/L (3.3 ± 1.04) on fasting and 5.9–12.6 mmol/L (8.58 ± 2.24) 2 h after meal were detected (Figure 3A). HbA1c (glycosylated hemoglobin, Type A1C) level is 5.2%, and serum electrolyte levels are normal. PET‐CT scan with 68Ga exendin‐4, simulating GLP‐1 (glucagon‐like peptide‐1), showed no signs of GLP‐1R positive tumors. The pituitary glucocorticoid axis, pituitary sex hormone axis, and pituitary growth hormone axis function normally. The OGTT, peptide C, and insulin‐releasing tests show normal insulin release and correlate with serum glucose levels (Table 1). However, relatively low insulin levels indicate suppression of secretion. The simultaneous spot testing of serum glucose, insulin, peptide C, IGF‐I (insulin‐like growth factor‐1), and *β*‐hydroxybutyric acid on fasting revealed extreme hypoglycemia with relatively low expressed hypoglycemic factors without ketosis. Additionally, spot testing of serum glucose, cortisol, growth hormone IGF‐I, and *β*‐hydroxybutyric acid before lunch showed no elevation of hypoglycemic hormone (Table 2). Serum insulin antibody or anti‐islet cell antibody was not detectable. Prior to surgery, hypoglycemia can be reduced through dietary intervention. Whipple′s criteria for diagnosing hypoglycemia were met.

**Figure 1:**
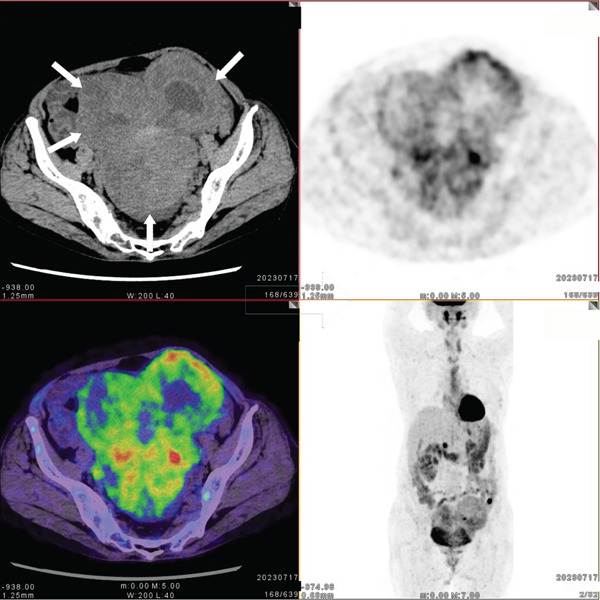
PET‐CT of the pelvic mass before primary surgery. Multiple cystic and solid masses are visible in the pelvic cavity (white arrows), with lobulated changes. The boundary between the local area and the posterior uterine wall serosa is indistinct, with the largest mass measuring approximately 10.7 × 10.0 × 10.0 cm in size. The SUVmax of the solid portion of FDG uptake increased to 7.7. No focal abnormalities were found in the lesion showing an increase in 68Ga exendin‐4 uptake.

**Figure 2:**
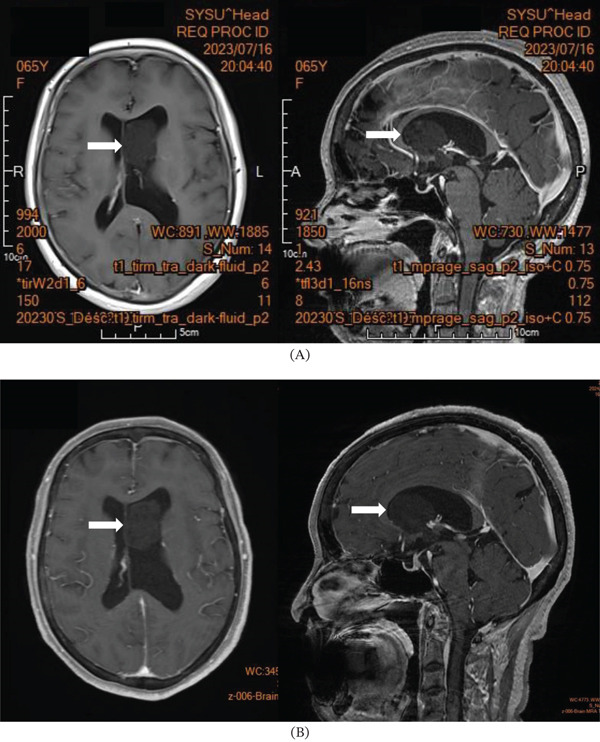
Head MRI examination. Nodule measuring 3.2 × 2.5 cm located in the anterior part of the left lateral ventricle, with low signal in T1WI, high signal in T2WI, and equal signal in T2 flair, and small nodular low signal inside considered to be a subependymal tumor (white arrows). (A) Image prior to operation for primary. (B) Image prior to operation for recurrence showed the same size of the nodule.

**Figure 3:**
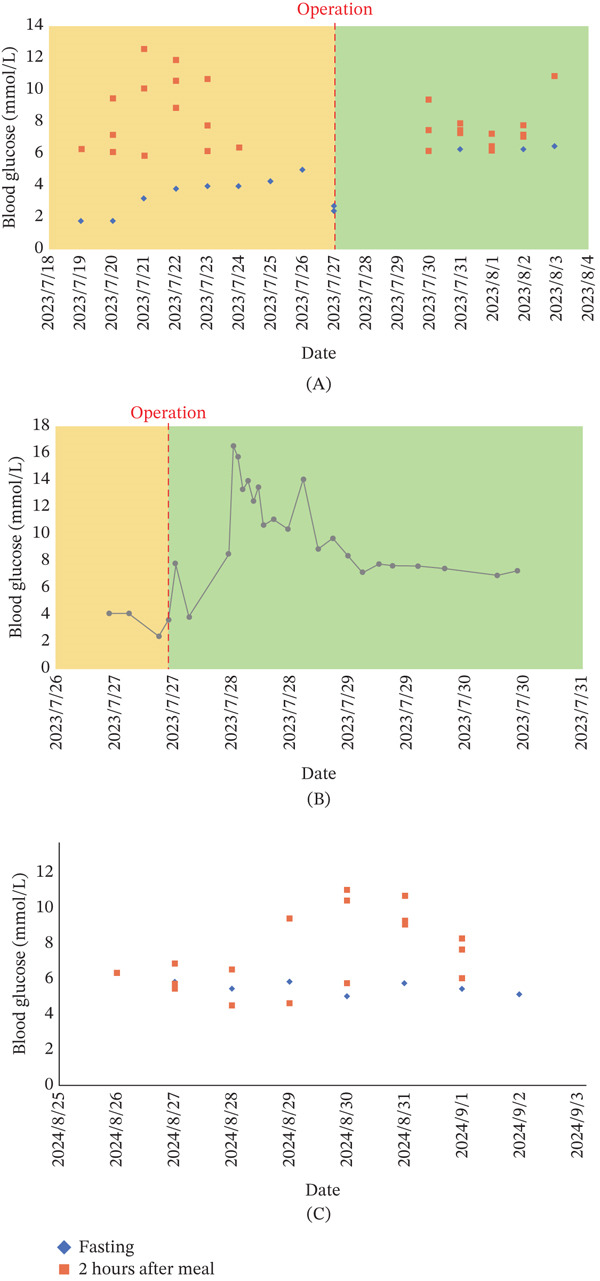
Blood glucose monitoring. Reference ranges for random blood glucose and for blood glucose 2 h after a meal are both 3.9–7.8 mmol/L, for fasting blood glucose is 3.9–6.0 mmol/L. (A) Prior to surgery, blood glucose monitoring showed lower fasting levels and a wide range of fluctuations. Postoperatively, blood glucose levels remained stable. (B) Preoperatively, blood glucose levels are low under intravenous drip of 5% glucose solution. While postoperatively, glucose levels can be elevated by intravenous drip of 5% glucose solution. (C) Blood glucose monitoring after recurrence showed normal on fasting and after meal.

**Table 1:** OGTT, peptide C, and insulin‐releasing tests.

	Fasting	1‐h	2‐h	3‐h	4‐h	5‐h
Insulin (uU/mL)	0.25	21.25	26.95	0.43	1.32	0.35
Peptide C (nmol/L)	0.036	0.993	1.35	0.192	0.436	0.145
OGTT (mmol/L)	3	10.1	9	2.6	3.1	2.7

**Table 2:** Tests for blood glucose regulation factors.

	2023/7/20	2023/7/20	2023/7/20	2023/7/21
	5:00 Fasting	8:00	12:00	12:00 Before meal
Insulin (uU/mL)	0.31			
Peptide C (nmol/L)	0.028			
IGF‐1 (ng/mL)	38.5			23.3
*β*‐hydroxybutyric acid (umol/L)	20.1			25.3
Blood glucose (mmol/L)	1.8			3.1
Cortisol (ug/dL)		5.5		5.5
Growth hormone (ug/L)				0.11
ACTH (pmol/L)		2.29	3.68	

Preoperative fasting caused hypoglycemia can be treated by intravenous administration of 5% glucose to raise capillary glucose level to 4–8 mmol/L. An abdominal total hysterectomy and bilateral salpingo‐oophorectomy were performed, and the en bloc resection of the tumors and uterus was realized (Figure 4). After surgery, intravenous administration of 5% glucose raised serum glucose levels to 10.6–16.7 mmol/L. (Figure 3B).

**Figure 4:**
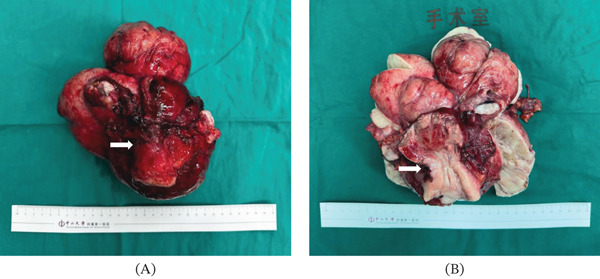
The gross appearance of the tumor. Uterine atrophy. (A) Several masses are generated from the uterine corpus. (B) The cut surface has a uniform appearance and is grayish‐yellow in color (white arrow for uterine corpus).

Postoperative pathology: The tumor cells were ovoid or polygonal in shape, with some arranged densely. The cytoplasm of the tumor cells is relatively small, light red stained, and the atypia is not obvious; mitotic figures were rare (2/10 high power fields [HPFs]), and multifocal fibrous collagen tissue can be seen in the tumor stroma, with multifocal infarction, tumor necrosis about 10%. It is preliminarily considered as a mesenchymal tumor. Immunohistochemistry staining: tumor cell CD99 (+), CD34 (+), a few cells desmin (+), individual focal CD10 (+), vimentin (+), P53 variable intensity (+), P16 scattered (+), Ki‐67 5% (+), cyclinD1 (−), actin/SMA (−), inhibin‐*α* (−), CK (−), EMA (−), CD31 (−), WT1 (−), PR (−), ER (−), H‐caldesmon (−), HMB‐45 (−), Melan‐A (−), CR(−), TFE‐3 (+), STAT‐6 (+), H3K27Me3 (+), SOX10 (−), S‐100 (−), Syn (−), CgA (−), and NTRK (−). Fluorescent in situ hybridization (FISH) detection for MDM2 gene amplification showed no amplification. The lesion was diagnosed as an epithelioid SFT generated from the uterine corpus (Figure 5). According to the 2020 WHO Classification (WHO Classification of Tumors: Soft Tissue and Bone Tumors. 5th ed) risk stratification for SFTs, this patient belonged to the intermediate risk group (Table 3).

**Figure 5:**
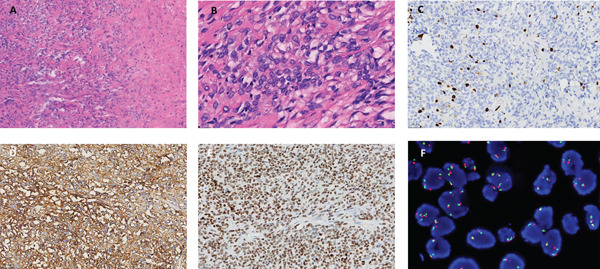
The results of the histopathology observation of primary surgery. (A) The tumor cells arranged in a sheet‐like pattern, with hyalinization with the stroma H&E staining 100×; (B) oval tumor cells with clear cytoplasm H&E staining 400×; (C) Ki‐67 indicated low proliferative activity 400×; (D) IHC demonstrating membranous staining of CD34 400×; (E) IHC demonstrating nuclear staining of STAT6 400×; and (F) the result of FISH indicating MDM2 gene amplification is negative.

**Table 3:** Risk stratification model from 2020 WHO Classification (WHO Classification of Tumors: Soft Tissue and Bone Tumors. 5th ed).

Risk factor	Cutoff	Points assigned	
		3‐variable model	4‐variable model
Patient age (year)	< 55	0	0
	≥ 55	1	1
Mitoses/mm^2^ (mitoses/10 HPFs)	0 (0)	0	0
	0.5–1.5 (1–3)	1	1
	≥ 2 (≥ 4)	2	2
Tumor size (cm)	0–4.9	0	0
	5–9.9	1	1
	10–14.9	2	2
	≥ 15	3	3
Tumor necrosis	< 10%	N/A	0
	≥ 10%	N/A	1
Risk	Low	0–2 points	0–3 points
	Intermediate	3–4 points	4–5 points
	High	5–6 points	6–7 points

Eight months after the initial treatment, PET‐CT found multiple soft tissue density mass shadows in the anterior sigmoid colon and posterior and superior bladder, the largest one was about 5.0 × 2.7 × 2.9 cm, considered SFTs (Figure 6). Considering patient has no obvious symptoms, we kept following up. Thirteen months after the operation, ultrasound scan showed several solid pelvic masses, the largest one was on the right side being about 93 × 58 mm and magnetic resonance imaging (MRI) showed the mass was partially poorly demarcated from the anterior wall of the upper rectum (Figure 7). MRI scan showed the anterior left lateral ventricle nodule was the same as before (Figure 2B). However, HbA1c 5.75% was checked, and fingertip blood glucose showed no hypoglycemia, which was 5–5.8 mmol/L (5.5 ± 0.3) on fasting and 4.5–10.9 mmol/L (7.4 ± 2.1) 2‐h postprandial (Figure 3C). Transabdominal tumor cytoreduction was performed, including omentectomy, partial rectal resection (resection of pelvic and abdominal metastatic lesions), and appendectomy. Postoperative pathology: Multiple tumor nodules in the rectal intestinal wall, tumor cells are diffusely distributed, ovoid or fat spindle shaped, epithelioid, nuclei are enlarged, irregular, mitosis is visible, part of the mesenchyme with vitreous degeneration, with visible necrosis. Immunohistochemistry staining: tumor cells CD34 (+), CD99 (+), Bcl‐2 (+), STAT‐6 (+), TFE‐3 (partially +), actin (−), CD117 (−), EMA (−), S‐100 (−), desmin (−), and Ki‐67 15% (+). The lesion was consistent with metastasis/recurrence of an epithelioid isolated fibrous tumor without definite nerve bundle invasion or intravascular tumor thrombus. The small bowel mesenteric mass, rectal surface mass, left pelvic wall peritoneal mass, appendiceal mass, left paracolic sulcus mass, left ovarian vein mass, left main ligament mass, and greater omental nodule are considered metastases/recurrences of SFT. The lesion was diagnosed as metastatic epithelioid SFTs (Figure 8).

**Figure 6:**
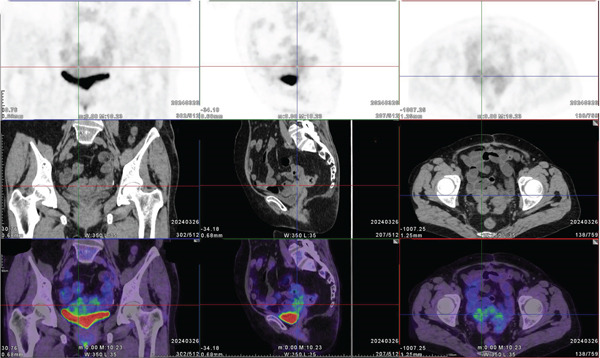
PET‐CT 8 months after the initial treatment. No abnormal density shadow or abnormal FDG uptake was seen in the vaginal stump. Multiple soft tissue density mass shadows were observed anterior to the sigmoid colon and superior to the posterior bladder, with the larger one measuring approximately 5.0 × 2.7 × 2.9 cm, with increased FDG uptake and a SUVmax of approximately 8.5. Solitary fibrous tumor was considered in conjunction with the preimaging, pathology, and history.

**Figure 7:**
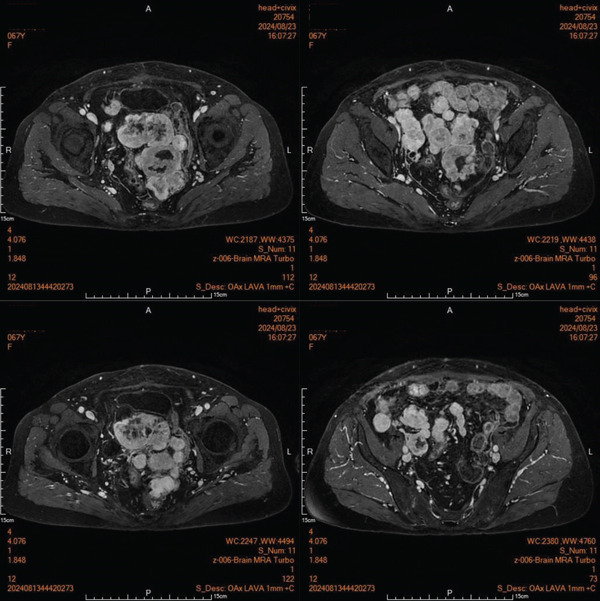
MRI 13 months after the initial treatment. The vaginal stump, the lower abdomen, and pelvis were seen to have multiple nodules/masses of varying sizes, the largest of which was about 49 × 44 mm, with poorly homogeneous signals, slightly high signal on T1WI, slightly high signal on T2WI, and some of the lesions with even higher signal, with high signals on high b‐value DWI, low signals on ADC, and enhanced scanning showed ring/homogeneous obviously enhancement. The localized mass was poorly demarcated from the anterior wall of the upper rectum; multiple effusion signal shadows were seen in the surrounding fat spaces. Consider tumor recurrence/metastasis.

**Figure 8:**
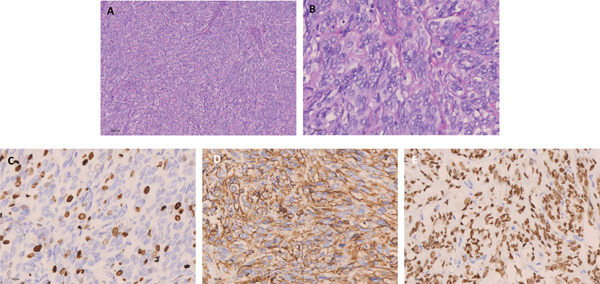
The results of the histopathology observation of recurrent surgery. (A) H&E staining 100×; (B) H&E staining of 400×; (C) Ki‐67 IHC staining 400×; (D) CD34 IHC staining of 400×; and (E) STAT6 IHC staining of 400×.

## 3. Discussion

SFTs generated from uterine corpus are rare and difficult to differentiate from leiomyomas without pathological diagnosis, because symptoms and signs are often similar based on the happening part and the size [2, 4]. Additionally, it is challenging to distinguish SFTs from other mesenchymal neoplasms using pelvic imaging techniques. Even when complicated by hypoglycemia, some patients may be asymptomatic. Because of the unsatisfactory therapeutic results of systemic therapy and radiotherapy, the main treatment of SFTs is complete surgical resection of the tumor. Therefore, comprehensive preoperative evaluation and selection of the correct surgical approach are important to improve the patient′s prognosis.

This case demonstrates the presence of hidden hypoglycemic symptoms with a large pelvic mass. It is necessary to exclude other potential causes of hypoglycemia, such as excessive secretion of endogenous hormones, deficiency of insulin antagonist hormone, or dysfunction of pancreatic islet *β* cells. 18F‐FDG and 68Ga exendin‐4 PET‐CT were both applied, 18F‐FDG uptake showed tumor hypermetabolism, and 68Ga exendin‐4 simulating GLP‐1 (glucagon‐like peptide‐1) whose uptake showed GLP‐1R positive tumors. The imaging indicates malignant uterine masses, but no signs of insulinoma or intracranial tumor, and the antibody against insulin is negative. The secretion of insulin antagonist hormones is normal, and both the oral glucose tolerance test and insulin release test show no abnormalities with pancreatic islet *β* cell function and insulin resistance. Additionally, imaging indicates a pelvic mass that may originate from the uterine corpus. Based on all the examinations, NITCH is considered.

Pathological diagnosis of SFT generated from uterine corpus is made after abdominal total hysterectomy and bilateral salpingo‐oophorectomy. In addition to HE staining, CD34 and/or STAT6 expression by IHC is essential for SFT diagnosis. NGFI‐A–binding protein 2‐signal transducer and activator of transcription 6 (NAB2‐STAT6) gene fusion is recommended to be a diagnostic marker for SFT [5]. STAT6 IHC staining is also reported to be suitable for SFT diagnosis, which is diffusely strong positive expression in nuclear in 90%–100% of cases, with high sensitivity and specificity [6]. As this tumor originates in the pelvis, a negative result of FISH detection for MDM2 gene amplification excluded adipose‐derived tumors [7].

In this case, the serum levels of blood glucose, insulin, IGF‐I, and *β*‐hydroxybutyric indicate that hypoglycemia may be caused by hypoglycemic factors other than insulin and IGF‐I; this leads to a diagnosis of NITCH or DPS [8]. Tumor larger than 20 cm or generated from pelvic/retroperitoneal is more likely to be complicated with DPS. The causes of NITCH are unclear. Possible reasons include the following: (1) mechanical compression of the autonomic nervous system causes excessive excitation of the vagus nerve; (2) increased tumor metabolism increases glucose consumption; (3) inhibition of gluconeogenesis; or (4) tumor secretes insulin‐like substances, mainly IGF‐I and IGF‐II. Research on DPS has shown that overexpression of high molecular weight (HMW) IGF‐II, the precursor of IGF‐II, by tumor cells may be the main cause of hypoglycemia. However, the serum IGF‐II level is an imperfect reflection of tumor‐secreted HMW IGF‐II, and IGF‐II IHC staining of tumor tissue reflects the secretion ability indirectly. Furthermore, IGF‐II can not only lower blood glucose directly but also inhibit the secretion of IGF‐I and IGF binding protein (IGFBP)‐3 in liver by inhibition of hypophysis GH releasing. Followed by reduction of IGF‐IGFBP‐3–Als, complex synthesis and releasing of free IGFs can cause hypoglycemia in the end [9, 10]. As a result, no clinically reliable indicators can be used to confirm the diagnosis of DPS.

Although SFTs are generally considered low‐grade malignant tumors, approximately 10%–40% of cases exhibit invasive and malignant processes. The 5‐year survival rates range from 59% to 100%, whereas the 10‐year survival rates range from 40% to 89%. Recurrence can occur even after 10 years of follow‐up [11–13]. It is important to note that tumors considered histologically benign may still relapse aggressively. The 2020 WHO classification proposes a risk stratification for SFTs, regardless of the histologically benign or malignant, dividing SFTs into low, intermediate, and high‐risk groups [14, 15]. Radical resection of the tumor is also a prognostic factor in addition to pathological risk factors, because there is currently no effective adjuvant systemic therapy for SFT. However, due to limited space in the pelvic cavity, it is difficult to perform a “surgical resection within the organ envelope” for a massive uterus, as is done for gastrointestinal tumors; moreover, compression during the procedure may increase the risk of tumor cell dissemination. Although radiotherapy has a certain effect on controlling local recurrence, SFT is mostly characterized by disseminated metastasis when recurrence [16]. This case underwent abdominal surgery without tumor residue and falls into the intermediate‐risk group, but there was recurrence 8 months after surgery, and the patient accepted tumor cytoreductive surgery at 13 months after initial treatment. Intraoperative exploration revealed disseminated, expansive growth of the tumor, and recurrent pathology confirmed the diagnosis of an SFT, which still showed no obvious signs of malignancy. However, it is noteworthy that the patient did not develop hypoglycemia upon recurrence even with a high tumor burden. There are reports of recurrent SFT complicated by DPS but without hypoglycemia at the initial treatment of the disease [17, 18]. Nevertheless, there is a shortage of relevant reports on the correlation between the occurrence of DPS and the condition of SFT diseases. The diagnosis of SFT occurring in the female reproductive system is difficult, and whether the presence of DPS has a guiding value in the diagnosis and treatment of this disease needs to be further investigated. Additionally, the study of the specific mechanism of SFT complicating DPS may help in the differential diagnosis of SFT and the early detection of tumor recurrence.

## Author Contributions

Lin‐jing Yuan and Ming Ni contributed equally to this work.

## Funding

No funding was received for this manuscript.

## Conflicts of Interest

The authors declare no conflicts of interest.

## Data Availability

Research data are not shared.
